# Preoperative Peak Oxygen Consumption: A Predictor of Survival in Resected Lung Cancer

**DOI:** 10.3390/cancers12040836

**Published:** 2020-03-31

**Authors:** Joerg Lindenmann, Nicole Fink-Neuboeck, Melanie Fediuk, Alfred Maier, Gabor Kovacs, Marija Balic, Josef Smolle, Freyja Maria Smolle-Juettner

**Affiliations:** 1 Division of Thoracic Surgery and Hyperbaric Surgery, Department of Surgery, Medical University of Graz, 8036 Graz, Austria; nicole.neuboeck@medunigraz.at (N.F.-N.); melanie.fediuk@medunigraz.at (M.F.); alf.maier@medunigraz.at (A.M.); freyja.smolle@medunigraz.at (F.M.S.-J.); 2 Division of Pulmonology, Department of Internal Medicine, Medical University of Graz, 8036 Graz, Austria; gabor.kovacs@medunigraz.at; 3 Ludwig Boltzmann Institute for Lung Vascular Research, 8036 Graz, Austria; 4 Division of Oncology, Department of Internal Medicine, Medical University of Graz, 8036 Graz, Austria; marija.balic@medunigraz.at; 5 Institute of Medical Informatics, Statistics and Documentation, Medical University of Graz, 8036 Graz, Austria; josef.smolle@medunigraz.at

**Keywords:** lung cancer, exercise capacity, preoperative peak, oxygen consumption, 10-year survival, prediction

## Abstract

The peak oxygen consumption (VO_2_ peak) serves as a prognostic factor in cardio-respiratory diseases and plays an important role in cancer patients. The long-term prognostic relevance of VO_2_ peak in lung cancer patients has not been investigated extensively. The aim of this study was to evaluate the impact of the preoperative VO_2_ peak on the postoperative long-term survival in patients with operated lung cancer. Retrospective analysis of 342 patients with curatively resected non-small-cell lung cancer using a multivariate Cox proportional hazard model. Results: Preoperative VO_2_ peak ranged from 10.2 to 51.8 mL/kg/min (mean: 18.3 ± 4.6), VO_2_ peak % of predicted ranged from 32 to 172% (mean: 65.2 ± 18.0%). Overall 10-year survival was 23%. A Log-rank test comparing predicted VO_2_ peak ≥ 60% with predicted VO_2_ peak < 60% showed overall survival of 30% and 17%, respectively (*p* < 0.001) and non-tumour-related survival of 71% and 51% (*p* = 0.001) at 10 years. In multivariable Cox analysis, overall 10-year survival correlated with a high predicted VO_2_ peak% (*p* = 0.001) and low N-stage corresponding to N0 and N1 (*p* < 0.001). Non-tumour-related death correlated with low VO_2_ peak% of predicted (*p* = 0.001), and age (*p* < 0.001). Low preoperative VO_2_ peak was associated with both decreased postoperative overall survival and decreased non-tumour-related survival during the 10-year follow-up.

## 1. Introduction

In spite of developments of chemo-, targeted-, immune- and radio-therapy, surgery still offers the best option for cure in lung cancer. Alongside moderately improving outcomes from lung resection [1], reports on long-term survival rates encompassing 10 years and more are emerging [2,3,4,5,6]. These studies focus on the prognostic influence of tumour stage and grade, tumour localisation, type of surgical resection and on epidemiologic data. Functional parameters are hardly referred to in the context of long-term survival of lung cancer, mainly being regarded as determinants for postoperative cardiopulmonary complications [7,8]. 

The peak oxygen consumption (VO_2_ peak), as assessed by cardiopulmonary exercise testing, reflects the maximum oxygen consumption for which association to various health outcomes has been documented [9]. 

It is a well-established prognostic factor in several cardiac and respiratory diseases [10,11,12] and has an important role in the planning of surgical therapy in patients with lung cancer [13,14]. In this context, it has been shown that preoperative short-term maximal cardiopulmonary exercise testing resulted in significant improvement in aerobic performances in patients awaiting lung cancer surgery [15]. Although the patient´s cardiorespiratory fitness could be enhanced perioperatively, long-term outcome and pulmonary function could not be improved at one year after surgery [16]. In particular, in those patients undergoing neoadjuvant chemotherapy, a significant decrease in both aerobic fitness and exercise capacity could be observed, resulting in a significantly reduced preoperative VO_2_ peak [17].

However, the long-term prognostic relevance of VO_2_ peak in lung cancer patients has not yet been investigated in detail, up to now. There are only few data available dealing with the clinical impact of preoperative VO_2_ peak on the postoperative survival in patients with curatively resected non-small-cell lung cancer (NSCLC) [18,19].

The aim of this study was to validate the prognostic impact of preoperatively evaluated VO_2_ peak in a large population of patients with curatively resected NSCLC. The correlation between predicted VO_2_ peak and the overall survival, the tumour-specific survival as well as the non-tumour-associated survival during a 10-year follow-up, including epidemiological and functional data, type of surgery as well as tumour-specific features, were assessed in a multivariable Cox proportional hazard model.

## 2. Results

The collective consisted of 225 males (65.8%) and 117 females (34.2%). We did 315 (92.1%) lobectomies or bi-lobectomies (thereof, 26 (7.6%) using sleeve-resection technique) and 27 (7.9%) pneumonectomies. 

Postoperative pathological staging yielded T1 in 186 (54.4%), T2 in 128 (37.4%), T3 in 18 (5.8%) and T4 in 7 (2%) cases. N0 was found in 191 (55,8%), N1 in 93 (27,2) and N2 in 58 (17%) patients. Union for International Cancer Control (UICC) staging revealed stage I in 182 patients (53.7 %), stage II in 85 patients (25.1 %) and stage III in 72 patients (21.2%). 43 patients had preoperative induction chemotherapy, resulting in complete pathological response in three of them. In 45 cases adjuvant chemotherapy, in 5 adjuvant chemo-radiotherapy and in 4 adjuvant radiotherapy was scheduled.

181 (53.2%) patients had chronic obstructive pulmonary disease (COPD). Coronary artery disease was present in 72 (21%) cases. Preoperative VO_2_ peak ranged from 10.2 to 51.8 mL/kg/min (mean: 18.3 ± 4.5), VO_2_ peak % of predicted ranged from 32 to 172 (mean: 65.2 ± 18.0). In 47.7% of patients VO_2_ peak was ≥ 60% of predicted, in 52.3% it was <60%. Forced Expiratory Pressure in 1 s (FEV_1_) ranged from 960 to 4650 mL/min (mean: 2422 mL/min ± 607.5) corresponding to a range of 36% to 151% of predicted (mean: 79.7 ± 16.5%). The patients´ characteristics are documented in detail in Table 1 and Table 2.

The median observation time for the total collective was 69.2 months (range: 0–184 months), whereas it amounted to 149.1 months for the survivors (range: 125–184 months). Overall 10 year survival rate was 23%. 157 patients died of lung cancer, 22 died of tumours other than bronchial carcinoma and 66 patients died of causes other than neoplasia, thereof 6 in the perioperative course (1.75%). The details are given in Table 3.

**Figure 1:**
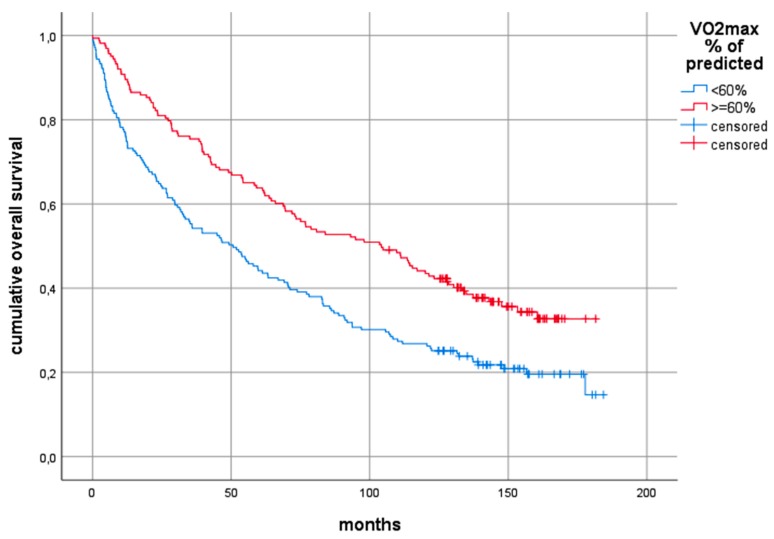
Kaplan–Meier curves comparing the overall 10-year survival between those patients with predicted VO_2_ peak >= 60% and those with predicted VO_2_ peak < 60%. Log-rank test: chi^2^ = 16.46, *p* < 0.0001. Abbreviations: VO_2_ peak: preoperative peak oxygen consumption.

**Figure 2:**
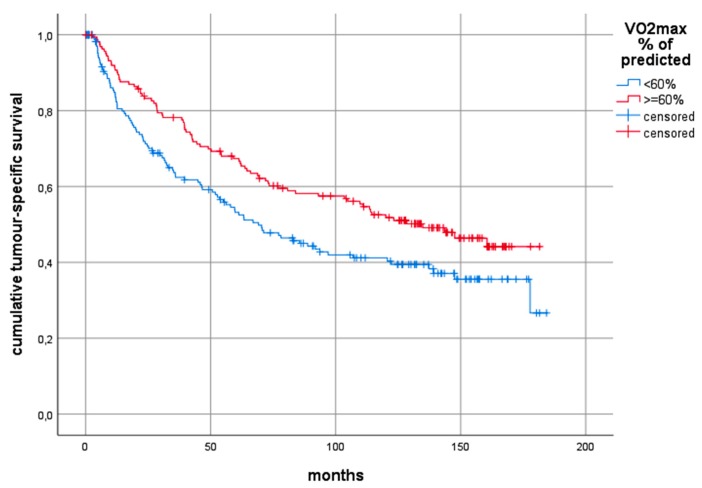
Kaplan–Meier curves comparing the tumour-related 10-year survival between those patients with predicted VO_2_ peak >= 60% and those with predicted VO_2_ peak < 60%. Log-rank test: chi^2^ = 7.87, *p* = 0.005. Abbreviations: VO_2_ peak: preoperative peak oxygen consumption.

**Figure 3:**
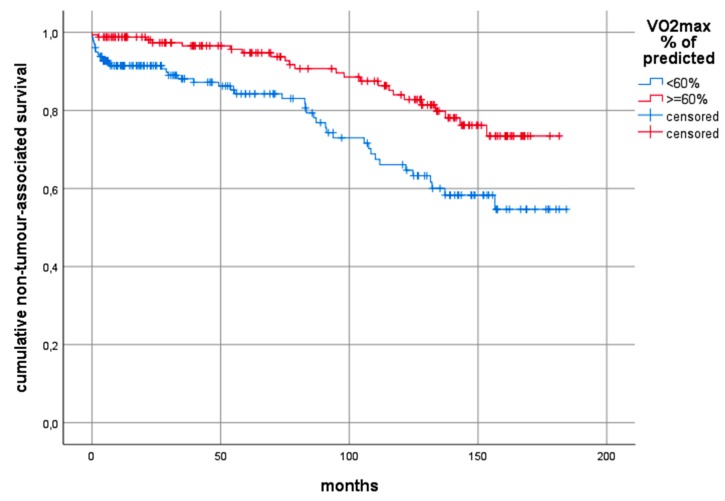
Kaplan–Meier curves comparing the non-tumour-related 10-year survival between those patients with predicted VO_2_ peak >= 60% and those with predicted VO_2_ peak < 60%. Log-rank test: chi^2^ = 10.78, *p* = 0.001. Abbreviations: VO_2_ peak: preoperative peak oxygen consumption.

The Log-rank test comparing predicted VO_2_ peak≥ 60% with predicted VO_2_ peak < 60% showed statistically significant differences with overall 10-year survival of 30% and 17% respectively (*p* < 0.0001), 10-year tumour-specific survival of 43% and 32% (*p* = 0.005) and 10-year non-tumour-related survival of 71% and 51% (*p* = 0.001). A split at 60% was chosen according to Brunelli [18]. Determining the best cut-point analysis in our cases using Classification and Regression Trees methodology (CART) based on the Cox proportional hazard model revealed similar values (58% for overall survival and tumour-specific survival, and 60% for non-tumour-related survival). The Kaplan–Meier curves based on the cut-point at 60% according to Brunelli [18] are shown in Figure 1, Figure 2 and Figure 3.

Peak oxygen consumption (VO_2_ peak of predicted) correlated positively with predicted FEV_1_, and negatively with ASA (ASA-Physical status; American Society of Anaesthesiologists) and, remarkably, postoperatively determined tumour invasion (pT) (Table 4). 

In univariable Cox analysis, VO_2_ peak of predicted was significantly associated with a more favourable outcome concerning overall survival, non-tumour-related survival and tumour-related survival (Table 5). 

In multivariable stepwise forward analysis (*p* = 0.1 for addition to the model), the VO_2_ peak entered the model of overall survival and non-tumour-related survival but was not significant in tumour-related survival. The other significant parameters and the corresponding hazard ratios are shown in Table 5 in the same order as they entered the model. 

Neo-adjuvant chemotherapy seemed to have a highly significant negative impact on overall and on tumour-related survival, most likely due to the unfavourable initial prognostic criteria of patients selected for this treatment modality. In addition, neo-adjuvant chemotherapy had a significant negative impact on the preoperative predicted VO_2_ peak. The mean of the predicted VO_2_ peak in these patients without neo-adjuvant chemotherapy was 66.3%, compared with 57.2% in those after neo-adjuvant chemotherapy (*p* = 0.0038). In contrast, neither adjuvant chemotherapy nor radiotherapy had a statistically significant impact on the VO_2_ peak (*p* = 0.7032 and *p* = 0.2738, respectively).

When the patients were split into three groups representing UICC stages I, II, and III, predicted VO_2_ peak was a significant prognostic parameter of overall survival in stage I and stage III, but not in stage II patients (univariable Cox analysis: *p* = 0.023, *p* = 0.534, and *p* = 0.002, respectively). Concerning non-tumour-related survival and tumour-related survival, predicted VO_2_ peak was only significant in stage III (*p* = 0.008 and *p* = 0.029, respectively). 

## 3. Discussion

This retrospective clinical study shows that low preoperative VO_2_ peak is associated with both decreased postoperative overall survival and decreased non-tumour-related survival during the 10-year follow-up. 

Peak oxygen consumption (VO_2_ peak) is a valid parameter predicting the risk of complications in the postoperative course following major surgery. Several studies have proposed cut-off points for the VO_2_ peak [7,20,21,22], adding information for algorithms enabling to decide whether patients with borderline lung function should undergo major thoracic surgery [23,24,25]. Training during rehabilitation programs has shown effectiveness in improving aerobic capacity in the preoperative setting [15], facilitating resection in patients initially deemed unfit [18,26].

However, the long-term prognostic relevance up to 10 years of VO_2_ peak in operated lung cancer patients has not yet been investigated in detail so far. There are only few data available focusing on the influence of the preoperative VO_2_ peak on the postoperative survival in patients with curatively resected NSCLC [18,19].

Yet in 2014, Brunelli et al. found evidence that the VO_2_ peak may predict not only the perioperative course of NSCLC but also long-term survival rates. In their sample, predicted VO_2_ peak above 60% was connected with both a higher 5-year overall and tumour-specific survival rate. The effect of VO_2_ peak on survival was independent of confounders, as documented by multivariable analysis [18]. 

Using the same cut-off value for VO_2_ peak, we were able to corroborate Brunelli´s findings and to add further important evidence resulting in increased informative value. However, one difference could be found in the number of investigated patients. In the present study, 342 subjects were included, whereas Brunelli and co-workers had included a considerably smaller number of only 157 patients. Furthermore, our sample of patients with curatively resected NSCLC was less homogeneous compared with Brunelli´s sample [18]. The present study included all stages eligible for resection and in consequence, also patients who had induction chemotherapy and adjuvant treatment regimens. Surgery comprised both lobectomy and pneumonectomy, whereas Brunelli and team operated early stage NSCLC by using lobectomy and sub-lobar resection. In addition, the present study has a considerably longer median follow-up as compared to the 40 months reported by Brunelli. At a median overall observation time of 69.2 months and a median observation time of 149 months for survivors, the positive prognostic effect of high VO_2_ peak or predicted VO_2_ peak% was found for both tumour-specific survival and for death from other causes, as shown by Log-rank testing. In this analysis, the overall 10-year survival probability for patients with predicted VO_2_ peak ≥ 60% was almost twice as high as for those with predicted VO_2_ peak < 60%, which was shown in Figure 1. Though, in multivariable testing, nodal status and T-stage also had a significant influence on long-term survival, the impact of predicted VO_2_ peak on both, the overall survival and non-tumour-related survival during 10-year follow-up, seemed to be an independent one, showing up with statistical significance, as displayed in Table 5.

The findings of the present study can be confirmed by Jones and co-workers in a similar setting with a comparable number of study participants. Among 398 patients with curatively resected stage I-III NSCLC, VO_2_ peak could be detected as a strong independent predictor of survival. Although their median follow-up time was nearly ten years shorter compared to that of the present study, they could demonstrate an inverse association between preoperative VO_2_ peak and all-cause mortality in patients with curatively resected NSCLC [19].

Up to now, there is no question that VO_2_ peak can be improved by training in healthy individuals [27]. It is unclear whether our patients with higher VO_2_ peak had the habit of routine exercise in the first place. In this context, the patient´s individual lung function combined with his personal fitness seems to play a pivotal role in the postoperative survival. 

Although the correlation between preoperative VO_2_ peak and long-term lung cancer survival seems robust, aerobic capacity may be a surrogate parameter influenced by a number of further hitherto unknown factors that may as well influence tumour growth [28]. What is more, it is not possible to conclude whether activity or fitness is more important for survival. The question of to what degree physical training can reduce recurrence or death rates resulting from lung cancer in defined subsets of patients has yet to be investigated. Even in these patients with preoperative high-intensity interval training before lung cancer surgery, the expected impact was modest, as previously described by Karenovics and co-workers. In the course of this randomised trial, the patients’ cardiorespiratory fitness could be increased perioperatively. However, this effect was not associated with better functional and clinical outcomes one year after surgery for lung cancer [16].

Nevertheless, preoperative aerobic fitness in lung cancer patients may be decreased considerably after neo-adjuvant chemotherapy by chemotherapy-induced reduction in pulmonary diffusion capacity or heart toxicity. Among a small sample of 34 patients with operable locally advanced NSCLC, the patients’ subgroup undergoing neo-adjuvant chemotherapy was significantly associated with lower preoperative VO_2_ peak, as compared to those without induction therapy [17]. However, we could corroborate this data in the present study. After administration of neo-adjuvant chemotherapy, the preoperative predicted VO_2_ peak was significantly lowered by 13 percent in the current analysis (*p* = 0.0038). In contrast, there was no statistically significant correlation between VO_2_ peak and adjuvant chemotherapy (*p* = 0.7032) and radiotherapy (*p* = 0.2738).

Yet, exercise capacity mirrored by VO_2_ peak need not only be the direct result of training: applying a multivariable testing model on data acquired for meta-analysis, Blair and co-workers found cardiorespiratory fitness to be strongly associated with mortality. The association for activity and health was, however, not significant. Hence, they suggested an influence of inborn components on fitness [29]. 

This may also apply to other parameters: Jones and colleagues did a prospective study in inoperable lung cancer patients with distant metastases. In addition to determining the exercise capacity by a 6-min-walking distance test, they used a questionnaire for self-assessment of exercise behaviour. Only the measured exercise capacity was proven as a significant predictor of survival in this cohort treated with palliative intent, whereas the self-reported exercise behaviour just reached borderline significance [30].

The recent Copenhagen Study conducted by Jensen and team puts the concerns about reverse causation between fitness and prognosis in perspective [9]. Cardiorespiratory fitness was initially determined by measuring VO_2_ peak in more than 5000 healthy men who were consistently followed-up for more than 40 years. VO_2_ peak was statistically significantly inversely associated with death from cancer and all-cause mortality. Even after exclusion of subjects dying within 20 years of study inclusion, the results remained robust. This suggests an only minimal influence of reverse causation [9]. 

Finally, there are some limitations in the present study which have to be mentioned. First, the study was retrospective, observational and conducted at a single institution. Second, due to the heterogeneous sample of this study, we cannot rule out the presence of some residual confounding by factors that were not included in the analysis due to not being collected during data ascertainment. Third, and therefore the most important aspect which should be stressed, is the investigated sample itself. This relatively large cohort of 342 patients involved all current potentially resectable tumour stages with the corresponding different kind of resections. Due to these facts, the heterogeneity of the sample was increased considerably compared with more homogeneous and smaller study groups, as used by Brunelli et al. [18]. 

Notably, in case of splitting the current collective into smaller groups, the subsequent findings may change accordingly after calculation of the statistics. In this context, the statistic power seemed to have weakened when the predicted VO_2_ peak has been correlated with survival according to the tumour stages I, II and III, respectively (Table 6). These astonishing findings are due to the small number of individuals within these three sub-groups. For this reason, these individual results have to be interpreted with caution. It is important not to draw any premature conclusions from this univariable sub-analysis before seeing the entire context of the present study. In contrast, in multivariable prognostic analysis using the Cox proportional hazard model including the total sample of 342 patients, a statistically significant correlation between predicted VO_2_ peak and both 10-year overall survival and non-tumour-related survival could be documented (Table 5). 

## 4. Materials and Methods

We did a retrospective analysis of 342 consecutive patients with NSCLC who had resection with curative intent between January 2003 and December 2007. The patient-specific data were collected prospectively in the database of our university hospital and retrospectively extracted for statistical evaluation. The study has been approved by the Local Ethics Committee of the Medical University of Graz, Austria (EK: 29–193 ex 16/17). As this is a retrospective non-intervention study, the institutional review board waived the need for written informed consent from the patients, consistent with national regulations. 

All patients had preoperative spirometry and symptom limited cardio-pulmonary exercise test. We used an electronically braked cycle ergometer for the exercise test, applying a ramp pattern increase of work rate. The test duration was 8 to 12 min. The setpoints of the measurement were determined according to the suggestions by Wasserman [31]. Testing was stopped if at least one of the following signs or symptoms evolved: Dyspnea, fatigue, blood-pressure increase beyond 220/120 mmHg, depression of the ST segment on the electrocardiogram greater than 2 mm in at least two adjacent leads or angina pectoris. VO_2_ peak was defined as the mean VO_2_ during the last 15 s of exercise. VO_2_ peak values were compared to predicted values according to Brunelli [18].

In case of FEV_1_ (Forced Expiratory Pressure in 1 s) lower than 70% of predicted or in case of VO_2_ peak below 10 mL/kg/min, we scheduled an additional quantitative ventilation-perfusion lung scan estimating the percentage of perfused or ventilated lung tissue in the affected lobe or lung, respectively. Criteria excluding resection were predicted as postoperative FEV_1_ below 900 mL and/or VO_2_ peak below 10 mL/kg/min. Evaluation of the diffusing capacity of the lung for carbon monoxide (DLCO) was done in the minority of the patients and therefore, the DLCO data was not taken into consideration for the current study. Furthermore, ASA surgical risk classification was assessed before surgery (ASA-Physical status; American Society of Anaesthesiologists).

Patients had lobectomy, bi-lobectomy, sleeve-resection or pneumonectomy with complete mediastinal lymph node dissection performed by Board-certified thoracic surgeons. Sub-lobar resections (wedge resections and segmental resections) have been shown to be connected with higher rates of postoperative tumour recurrence and decreased survival compared with standard resection procedures and were therefore excluded from this retrospective evaluation [32,33,34]. Tumour staging was done according to the current tumour-node-metastasis (TNM) classification defined by the Union for International Cancer Control (UICC International Union Against Cancer, TNM Classification of Malignant Tumours, 8th edition) [35]. 

If possible, patients were extubated in the operating room. As a routine, they were admitted to the intensive-care unit for the first 24 h. Further care included early mobilisation, physiotherapy, inhalation and standardised analgetic therapy. Visual analogue scale scores (VAS-scores) were assessed four times a day in rest and in motion. Pain medication adhered to a certified, stepwise, guideline-based schedule [36], applying epidural analgesia whenever possible, enabling patient-controlled analgesia (PCEA). VAS ≤ 3 was considered as sufficient pain relief. 

### 4.1. Follow-Up and Data Management

The required follow-up data were retrieved from the Regional Health Care System database (open MEDOCS). All cases were followed-up through February 2019 or until death. However, if a patient had not shown up for follow-up, the respective family doctor was contacted for information. If the patient was suspected deceased, we did a data query at the Austrian Central Obituary Column. The causes of death were recorded. No patient was lost to follow-up. 

This meticulous procedure ensured a consistent follow-up, which allowed for at least a 10-year observation period for the sample. Overall survival was defined as the time from the date of surgery to the date of death-from-any-cause. Tumour-specific survival was determined from the date of surgery to the date of death after tumour recurrence. Non-tumour-associated survival was similarly computed from the date of surgery to the date of death from causes other than due the tumour recurrence.

### 4.2. Statistical Analysis

Statistical workup was performed using the SPSS 25 (Microsoft Inc., Chicago, Ill., USA) and STATA (Stata Corp., College Station, TX, USA) program package. Absolute- and relative-frequencies, mean values and standard deviation and median, minimum and maximum were used as descriptive statistics. Univariable survival analysis was performed with Kaplan–Maier survival curves and the Mantel–Haenszel Log-rank test. For multivariable analysis, the Cox proportional hazard model was applied in a stepwise forward procedure, with a significance level for addition to the model of 0.1. Correlation between various parameters was assessed by Spearman’s rank correlation test. To determine a best cut-point for predicted VO_2_ peak, CART (classification and regression tree) analysis based on the Cox proportional hazard model was applied. 

## 5. Conclusions

Regarding the findings of the present study, we may conclude that low preoperative VO_2_ peak is associated with both decreased postoperative overall survival and decreased non-tumour-related survival during a 10-year follow-up. In this context, the preoperative predicted VO_2_ peak may serve as a prognostic tool for survival in patients with curatively resected NSCLC. For this reason, it might be beneficial to take the preoperative predicted VO_2_ peak into consideration together with the postoperatively completed tumour stage for scheduling further treatment. Thus, larger prospective multicentric studies will have to be carried out in order to approve these preliminary results.

## Figures and Tables

**Table 1:** Characteristics of 342 patients undergoing curative resection for non-small cell lung cancer (part one). Abbreviations: BMI: Body Mass Index, FEV_1_: Forced Expiratory Pressure in 1 s, ml: milliliter, min: minute, VO_2_ peak: preoperative peak oxygen consumption, kg: kilogram body weight.

Patients Characteristics I	Mean ± SD	Range
Age	63.6 ± 9.4	37–86
BMI	25.9 ± 3.9	14.8–38.2
FEV_1 mL_/min	2422 ± 607.5	960–4650
FEV_1_ % of predicted	79.7 ± 16.5	36–151
VO_2_ peak ml/kg/min	18.3 ± 4.6	9.2–51.8
VO_2_ peak % of predicted	65.2 ± 18.0	32–172
Median observation time overall (months)	69.2	0–184
Median observation time survivors (months)	149.1	125–184

**Table 2:** Characteristics of 342 patients undergoing curative resection for non-small cell lung cancer (part two). Abbreviations: COPD: Chronic obstructive pulmonary disease, NSCLC: non-small cell lung cancer, T: T stage (tumour invasion), N: N stage (nodal involvement).

Patients Characteristics II	Number	Percentage (%)
COPD	181	52.9
Coronary artery disease	72	21.1
Lobectomy, Bi-Lobectomy	315	92.1
Pneumonectomy	27	7.9
Squamous Cell Carcinoma	112	32.7
Adenocarcinoma	140	40.9
Other type of NSCLC (large-cell, polymorphic, spindle-cell)	90	26.3
T0	3	0.9
T1	186	54.4
T2	128	37.4
T3	18	5.3
T4	7	2
N0	191	55.8
N1	93	27.2
N2	58	17
Induction Chemotherapy	43	12.6
Adjuvant Chemotherapy	45	13.1
Adjuvant Chemo-Radiotherapy	5	1.4
Adjuvant Radiotherapy	4	1.1

**Table 3:** Causes of death of those 245 patients undergoing curative resection for non-small-cell lung cancer.

Cause of Death	Number	Percentage (%)
Lung cancer	157	45.9
Neoplasia other than lung cancer	22	6.4
Other than neoplasia	66	19.3
Chronic cardiac failure	11	3.2
Pneumonia	9	2.6
Myocardial infarction	8	2.3
Renal failure	7	2.0
Stroke	7	2.0
Dementia	5	1.5
Right heart failure	5	1.5
COPD	3	0.9
Decrepitude	2	0.6
Pulmonary embolism	2	0.6
Ileus	2	0.6
Parkinson´s disease	1	0.3
Antibody deficiency syndrome	1	0.3
Multiorgan failure	1	0.3
Peritonitis	1	0.3
Influenza	1	0.3
Total	245	71.6

Abbreviation: COPD: Chronic obstructive pulmonary disease.

**Table 4:** Correlation of peak oxygen consumption (VO_2_ peak of predicted) with other parameters (n = 253; Spearman’s rank correlation test).

Parameter	Rho	*p*-Value
FEV_1_ of predicted	0.2923	< 0.0001
tumour diameter	−0.0912	0.1483
ASA	−0.2532	< 0.0001
grading	−0.0977	0.1212
pT	−0.1709	0.0064
pN	−0.1229	0.0509
margin	−0.1129	0.0731
age	0.0783	0.2143

Abbreviations: FEV_1_: Forced Expiratory Pressure in 1 s, ASA: ASA-Physical status (American Society of Anesthesiologists), T:T stage (tumour invasion), N:N stage (nodal involvement).

**Table 5:** Univariable and multivariable prognostic analysis using Cox’ proportional hazard model.

Criterion	Hazard Ratio	Std. Err.	Z	*p* > |z|	95% Conf. Interval	
10 year overall survival						
*univariable*						
VO_2_ peak	0.959	0.016	−2.48	0.013	0.928	0.991
VO_2_ peak of predicted	0.985	0.004	−3.50	0.000	0.976	0.993
*multivariable stepwise*						
pN	1.487	0.159	3.71	0.000	1.205	1.835
VO_2_ peak of predicted	0.983	0.004	−3.33	0.001	0.974	0.993
Age	1.029	0.009	3.32	0.001	1.012	1.047
Neo−adjuvant chemotherapy	2.250	0.529	3.45	0.001	1.418	3.569
Tumour diameter	1.087	0.054	1.69	0.091	0.986	1.199
10 year non-tumour-related survival						
*univariable*						
VO_2_ peak	0.894	0.033	−2.94	0.003	0.830	0.963
VO_2_ peak of predicted	0.973	0.008	−2.96	0.003	0.955	0.990
*multivariable stepwise*						
Age	1.065	0.018	3.61	0.000	1.029	1.102
VO_2_ peak of predicted	0.964	0.010	−3.36	0.001	0.944	0.985
10 year tumour-related survival						
*univariable*						
VO_2_ peak	0.975	0.017	−1.37	0.171	0.940	1.010
VO_2_ peak of predicted	0.988	0.004	−2.37	0.018	0.979	0.998
*multivariable stepwise*						
pN	1.558	0.191	3.61	0.000	1.224	1.984
Neo-adjuvant chemotherapy	2.875	0.710	4.28	0.000	1.772	4.666
Tumour diameter	1.171	0.063	2.92	0.003	1.053	1.301
Resection margin	1.976	0.856	1.57	0.116	0.844	4.622
Pneumonectomy	0.430	0.198	−1.82	0.068	0.174	1.064
Female gender	0.725	0.136	−1.71	0.087	0.501	1.048

Abbreviations: VO_2_ peak: preoperative peak oxygen consumption, N:N stage (nodal involvement).

**Table 6:** Univariable prognostic analysis of predicted VO_2_ peak using Cox proportional hazard model, evaluated separately for Union for International Cancer Control (UICC) stages I, II and III.

Impact of Predicted VO_2_ Peak	Hazard Ratio	Std. Err.	z	*p* > |z|	95% Conf. Interval	
10-year overall survival						
UICC I	0.985	0.006	−2.28	0.023	0.973	0.997
UICC II	0.995	0.006	−0.62	0.534	0.982	1.009
UICC III	0.966	0.010	−3.09	0.002	0.945	0.987
10-year non-tumour-related survival						
UICC I	0.982	0.011	−1.60	0.110	0.960	1.004
UICC II	0.973	0.017	−1.50	0.135	0.940	1.008
UICC III	0.893	0.037	−2.67	0.008	0.821	0.970
10-year tumour-related survival						
UICC I	0.986	0.007	−1.82	0.068	0.971	1.001
UICC II	1.000	0.007	0.12	0.907	0.987	1.014
UICC III	0.975	0.010	−2.18	0.029	0.954	0.997

Abbreviations: VO_2_ peak: preoperative peak oxygen consumption.
